# Mechanical determinants of chromatin topology and gene expression

**DOI:** 10.1080/19491034.2022.2038868

**Published:** 2022-02-27

**Authors:** Rajiv Kumar Jha, David Levens, Fedor Kouzine

**Affiliations:** Gene Regulation Section, Laboratory of Pathology, Nci/nih, Bethesda, MD USA

**Keywords:** Transcription, chromatin, psoralen, DNA supercoiling, 3D genome, topoisomerase, loop extrusion, cohesin, cell cycle, epigenetics

## Abstract

The compaction of linear DNA into micrometer-sized nuclear boundaries involves the establishment of specific three-dimensional (3D) DNA structures complexed with histone proteins that form chromatin. The resulting structures modulate essential nuclear processes such as transcription, replication, and repair to facilitate or impede their multi-step progression and these contribute to dynamic modification of the 3D-genome organization. It is generally accepted that protein–protein and protein–DNA interactions form the basis of 3D-genome organization. However, the constant generation of mechanical forces, torques, and other stresses produced by various proteins translocating along DNA could be playing a larger role in genome organization than currently appreciated. Clearly, a thorough understanding of the mechanical determinants imposed by DNA transactions on the 3D organization of the genome is required. We provide here an overview of our current knowledge and highlight the importance of DNA and chromatin mechanics in gene expression.

## Overview

### Basics of three-dimensional genome organization

The mechanical contributions of genome folding into gene regulation are multi-layered involving: DNA base pairing, nucleosome formation, nucleosome organization, chromatin loops, and the formation of Topologically Associating Domains (TAD) [1].

(1) DNA base-pairing: DNA sequences determine DNA base pairing. The disruption of DNA base pairing occurs during processes where the genetic information is copied into an RNA (transcription) or a DNA molecule (replication). Special DNA sequences are also prone to form non-B DNA structures that are functionally important in a variety of physiological and pathological conditions [2]. A prerequisite for the formation of these structures is duplex destabilization. Finally, recognition and binding of regulatory factors depends on local DNA structure. To implement these regulatory events, the stability and stiffness of the DNA double helix might be overcome by forces and torques applied to DNA and chromatin.

(2) Nucleosome formation: The basic unit of chromatin is a nucleosome which consists of ~147 bp of core DNA wrapped nearly two times around a conserved histone-octamer composed of H2A, H2B, H3, and H4 [3]. Nucleosome formation is an important regulatory event in genomic processes as it interferes with protein factors binding to DNA. The affinity of the histones to DNA is assumed to provide the energy required to wrap DNA around the core histones [4]. Various biochemical assays and computational modeling have shown that nucleosomes are dynamic in nature and exhibit robust response to mechanical stimuli. Thus, the contribution from forces applied to the DNA might supplement or oppose the energy of histone–DNA interactions.

(3) Nucleosome organization: Individual nucleosomes are separated by a short linker DNA, giving the classical 10 nm ‘beads-on-a-string’ appearance. Early *in vitro* studies under high salt concentrations showed a transition of 10 nm to 30-nm condensed structures. However, recent experiments argue against the existence of these 30 nm structures *in vivo*. Super-resolution and live-cell imaging revealed an irregular transition from nucleosome free regions to 10-nm structures and to clusters of nucleosomes with different degrees of compaction [5]. The interactions between distantly spaced genomic segments are influenced by the nucleosome organization.

(4) Chromatin loop: The next level of chromatin folding is loop-formation, considered as a ubiquitous feature in the regulation of genomic transactions. The base of loops display increased chromosomal contact between separated genomic regions, perhaps established by protein(s) bound on one side of the loop bridging with protein(s) bound to the other [6]. Loops ranging in size of tens kbs often bring enhancers to the targeted promoters [7]. The regulatory specificity of enhancers is thought to be controlled by their sequence and by the binding of transcription factors. In the current models, loop formation is not a spontaneous process but demands energy to bring interacting partners together.

(5) Topologically Associating Domains (TADs) formation: Development of chromosome conformation capture-based techniques (Hi-C) has shown that at a sub-megabase scale, chromatin is folded into TADs [8]. On a population level, each TAD is characterized by a relatively high number of genomic contacts. TADs preferentially encompass chromatin loops established by interaction between enhancers and gene promoters, and so confine the zone of enhancer action. Currently, the most popular model of TADs formation is loop extrusion driven by cohesin proteins and stabilized by CCCTC-binding factor (CTCF) [9]. The importance of this mechanism has been questioned by recent super-resolution imaging experiments, which found that domain boundaries are highly stochastic at the single-cell level [10,11].

Changes in genome organization at each of these levels have been linked to functional changes suggesting an intimate role of 3D-chromatin structure in genome function [1,6]. Here, we aim to emphasize that reverse is also true: those genomic transactions strongly influence genome folding and organization.

### Basics of DNA mechanics

**Figure 1.**
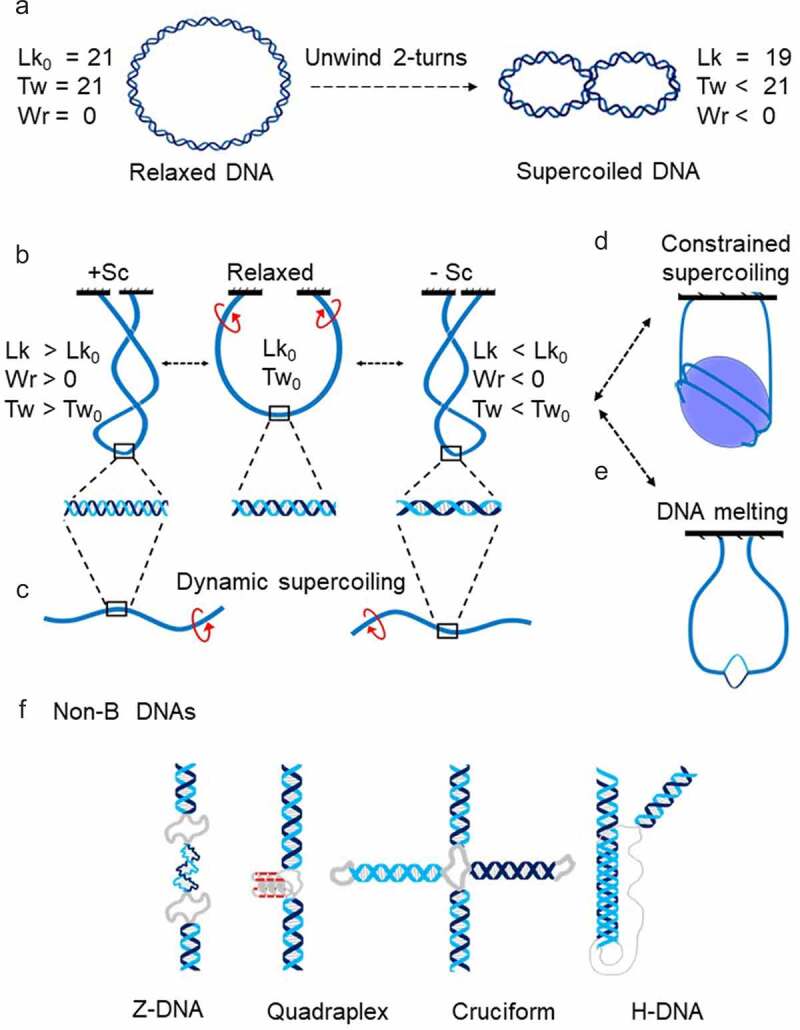
**Basic of DNA Supercoiling**. DNA supercoiling is a physical property of the DNA double helix [12], usually ascribed to circular DNA, such as plasmids. Ligation of ends of a linear DNA results in a unconstrained planar circle of duplex DNA made of two strands. Two strands of circular DNA are interlinked and the number of interlinks is called the linking number (LK). LK can only change if one or both DNA strands are transiently cut. The linking number of ‘relaxed’ DNA (Lk_0_) reflects the geometry of the double helix: each 10.5 bp of the helical repeat produces one interlink. A relaxed circular DNA with 21 helical turns has Lk = 21. Lk is a function of the twist (Tw) and writhe (Wr): Lk = Tw+Wr. In the first approximation, twist is a measure of the winding of DNA strands around each other. Therefore, for relaxed DNA shown in this figure Lk_0_ = Tw_0_ = 21 (**A**, left). Because double helix resists bending and twisting, changing the Tw is compensated by coiling of the double helix axis which is measured by Wr. If the Twist number is altered before ligation, the DNA molecule adopts a supercoiled conformation (**A**, right). Topologically, immobilizing the end of DNA fragment fixes the number of links between the two DNA strands, mirroring the ligation of DNA fragment to form a circle. Thus, supercoiling can also be imposed on topologically constrained noncircular DNA molecules (b). Negative supercoils (-Sc) is generated by un-twisting (**B**, right), while positive supercoils (+Sc) is due to over-twisting of double helix (**B**, left). Supercoiled DNA molecule is under torsional stress. Accordingly, transient propagation of torsional stress along the DNA axis away from its mechanical source results in dynamic supercoiling (c). Although the Lk in topologically constrained DNA cannot be changed without breaking DNA strands, several processes alter distribution of torsional stress along the molecule. Writing part of supercoiling can be manifested as plectoneme, or as toroid when constrained in a nucleosome (d). Constrained supercoiling does not impose torsional stress on adjacent regions until liberated. Torsional stress in negatively supercoiled DNA promotes strand-separation and can be released by formation of melted DNA bubble (e) or by formation of other non-B DNA structures (f). These structures form on tracts of low complexity sequences that are abundant in genomes and occurs at specific genomic locations, supporting a functional role of non-B DNA structures in genomic transactions [2].

**Figure 2.**
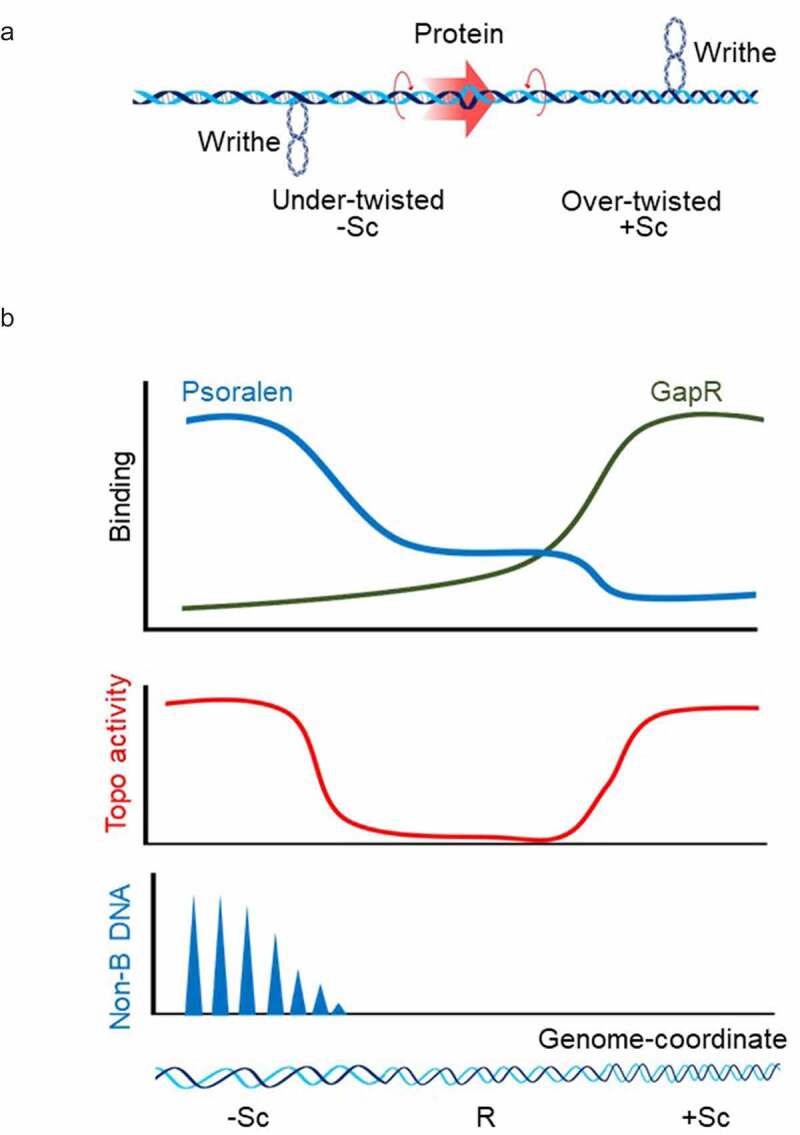
**DNA Supercoiling *in vivo*** (a) Twin-supercoiled domain model explaining the generation of dynamic positive supercoils ahead and negative supercoils behind of the protein complex that translocates along the DNA double helix [15]. Although this supercoiling regulates the variety of DNA transactions, excessive DNA supercoils will halt the further progression of translocating complex if not properly resolved. (b) Methods for detection of DNA supercoiling *in vivo*. Top panel: DNA supercoiling have been most frequently probed with psoralen. Psoralen freely crosses cellular membranes, intercalates between DNA bases and forms crosslinks between the two strands when exposed to UV light [127]. It has a different preference for relaxed, positively supercoiled, and negatively supercoiled DNA (blue curve). Taking advantage of this psoralen property, supercoiled DNA have been mapped in bacteria, yeast, Drosophila, and human cells [66,69,119,145]. Recently developed GapR-seq assay is based on the ability of the bacterial protein GapR to preferentially recognize overtwisted DNA (green curve). Chromatin immunoprecipitation of GapR combined with high-throughput sequencing was used to generate maps of positive supercoiling in bacteria and yeast [97]. Detection of topoisomerase activity sites (Middle panel) and non-B DNA structures (Bottom panel) are also powerful methods to predict DNA supercoiling *in vivo* [71,132,146]. There has been considerable concordance between the studies supporting the main prophecies of the twin-supercoiled domain model: negative torsional stress accumulated at the upstream promoter region of the active genes, while positive torsional stress accrues in a transcription-dependent manner in gene bodies and downstream to the 3’ ends of genes. – Sc (negatively supercoiled DNA); R (relaxed DNA); +Sc (positively supercoiled DNA). Blue triangle (Non-B DNA).

DNA can be regarded as a physical object that is subject to various forces in a chromatin environment. All genomes are constantly remodeled by proteins acting on DNA that generate torsion and apply tension on the double helix [12]. The best studied mechanism for generating torsion is the progressive motion of protein complexes translocating on DNA. In particular, elongating RNA polymerase tracks along the right-handed DNA double helix causing axial rotation of the transcribed DNA relative to the polymerase. The ‘twin-supercoiled domain model’ proposes that hindering the free rotation of DNA ends causes the double helix to become over-twisted in front of the polymerase, and under-twisted behind the polymerase, leading to torsional stress [13]. Geometrical parameters of the double helix (overtwisting/undertwisting and associated coiling of the axis of the double helix) are referred as DNA supercoiling (Figure 1). The original model suggested that accumulation of torsional stress might occur under conditions that prevented the free rotation of DNA, or in the absence of DNA topoisomerases. DNA topoisomerases are enzymes that remove torsional stress by transiently breaking and resealing DNA strands [14]. About a decade later, reevaluation of this model suggested that transcription can induce significant torsional stress even in linear unanchored DNA and in the presence of active DNA topoisomerases (Figure 2**, A**) [15–17]. Finally, a variety of biochemical studies, single-molecule experiments, and *in vivo* assays allowed careful characterization of transcriptionally generated torsion (Figure 2**, B**) and suggested its importance in genomic transactions [18–21]. The supercoiled domain model applies with minor modification to all activities that force DNA to revolve around its axis such as movement of replisome, helicases, and type I restriction enzyme activity [22]. DNA loop extruding enzymes might generate torsional stress inside the loop that is topologically isolated from the rest of the DNA. Selective DNA relaxation in the loop by topoisomerases would then yield a net supercoiling across the genome after loop dismantlement [23,24].

DNA is supercoiled as a result of coiling its axis around core histones on the nucleosome in a left-handed direction [3]. However, this supercoiling is constrained by DNA–protein interactions and cannot be resolved by action of DNA topoisomerase until released from the nucleosome (Figure 1). Chromatin remodeler complexes modify, slide, or remove nucleosomes [24]. Such reorganization of eukaryotic chromatin releases negative DNA supercoiling [25]. In addition, *in vitro* experiments show that in the process of nucleosome destabilization, the chromatin remodelers might generally and directly introduce negative torsional stress into the DNA [26]. It should be noted that transcription continuously generates DNA supercoiling at a much higher rate in comparison with one-step removal of nucleosomes. Consequently, *in vivo* confirmation of the functional role of DNA supercoiling generated during nucleosome reorganization is scarce and largely qualitative [27,28]. The intimate relationship between nucleosome structure and supercoiling indicates that DNA torsional stress has a strong impact on nucleosome structure and stability [19].

## Step by step transcription

The transcriptional machinery moves to productive RNA elongation through multiple steps: nucleosome remodeling at the promoters; transcription factor binding; RNA Polymerase II (Pol II) recruitment; melting of DNA during open promoter complex formation; promoter clearance; promoter-proximal pausing of Pol II; pause release and RNA elongation [29]. Gene expression is the outcome of regulation at each of these steps as well as promoter–enhancer interactions and TAD formation [6,29]. Key to understand gene expression is an in-depth knowledge of how chromatin structure can be dynamically reorganized [1]. Transcriptional control in prokaryotes and eukaryotes is markedly different because the eukaryotic genome is packed into chromatin [30,31]. This difference is essential for the diverse pattern of eukaryotic gene expression. While prokaryotic cells possess many analogous mechanisms that translates mechanical stimuli on DNA to gene regulation [32,33], we put aside the bacteria kingdom in our review. Here, our goal is to demonstrate that DNA mechanical constraints introduced into the chromatin by transcription work as a feedback mechanism to regulate gene expression across multiple levels of 3D genome organization.

### Transcription factors binding and pol II recruitment

Early plasmid-based experiments showed that negatively supercoiled DNA yields higher levels of gene expression than relaxed, suggesting that this topological state creates a favorable environment for general transcription factors (TFs) and RNA polymerases [34–36]. *In vitro* transcription performed with a minimal set of factors showed that DNA supercoiling facilitates the binding of transcription factors TFIID and TATA binding protein (TBP) to the promoter [34,35]. Importantly, TBP is a key component of the transcription initiation machinery, considered as a major interaction hub within the preinitiation complex (PIC) [37]. The dissection of the different steps in transcription reveals that DNA supercoiling promotes DNA melting and consequent formation of an open complex for transcription initiation [36]. The highly specific binding of TFs to their corresponding DNA targets is established by the direct readout of the target sequence as well as by the geometry of the double helix. The shape of the DNA constitutes, in fact, a second constraint recognized through shape readout mechanisms [38,39]. Evidently, DNA supercoiling changes the geometry of the double helix modulating the affinity of certain TFs to DNA. Conversely, TF binding by itself has the capacity to modify DNA response to supercoiling and modulate the affinity of other factors for their targets on the promoter [39,40]. Although it is well established that TF-DNA interactions is key to transcriptional control in eukaryotic cells, our understanding of the mechanistic and dynamic aspects of these interactions is still somewhat rudimentary.

Promising advances in technology have enhanced our understanding of the role of DNA supercoiling in the specific targeting of TFs to the chromatin. A recent genome-wide study has compared the occupancy of various chromatin-binding proteins (by ChIP-seq) and DNA supercoiling map obtained by psoralen intercalation analysis (TMP-seq) [41]. From a cohort of 10 proteins with different functions, all 4 transcriptional factors Fos, Jun, JunB1 and Satb1 have been shown to have sharp preference for localization on under-twisted, negatively supercoiled DNA. This result might represent only the tip of the iceberg and the dependence of DNA recognition by regulatory factors on mechano-sensors might be much broader than we imagine. Of note is also the discovery of very high conformational diversity of individual negatively supercoiled DNA minicircles by high resolution atomic force microscopy and molecular dynamics simulations [42]. This diversity is proposed to allow many structural perturbations which could better accommodate the binding of molecular partner. In addition, a cooperative effect between global DNA conformation and molecular recognition of the short sequences has been observed [42], which clearly indicates how information from the binding of a TF can be transferred through DNA to a distal TF by the imposition of torsional stress through double helix.

**Figure 3.**
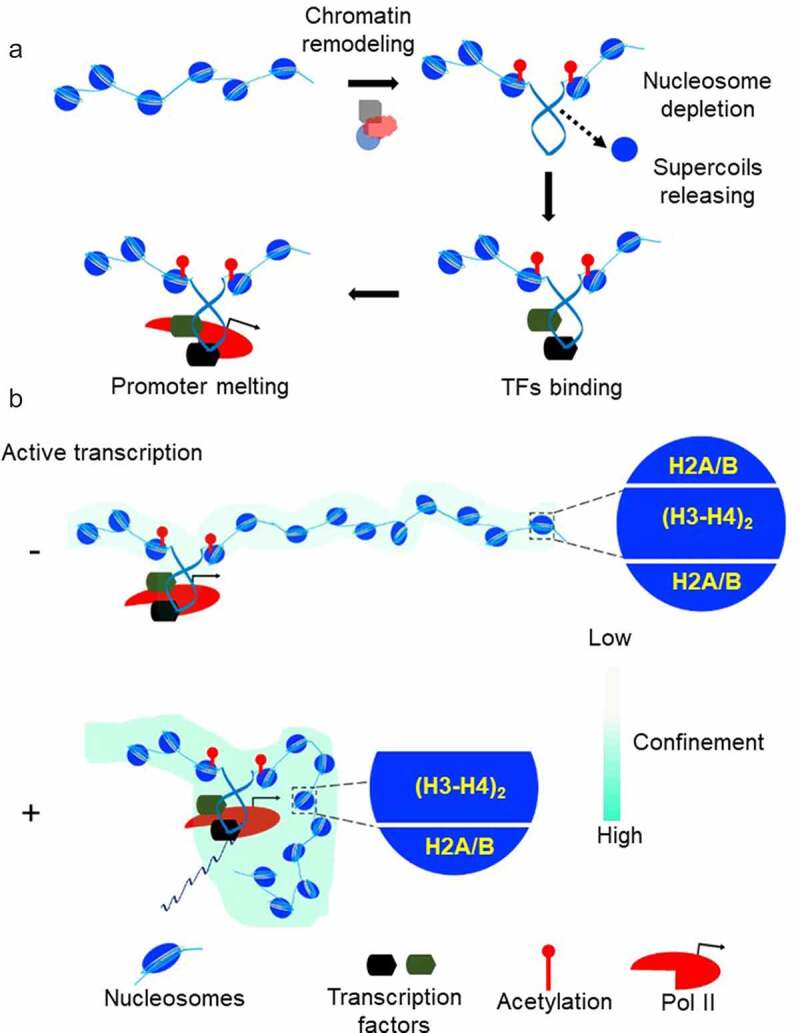
**Chromatin mechanics and gene expression** (a) The pre-initiation complex formation often involves the recruitment of chromatin remodeling complexes and histone acetyltransferases on the promoter. Core histone rearrangement and/or acetylation release negative supercoils previously constrained by the nucleosomes. Negative supercoiling increases affinity of TFs to promoter DNA, helps recruitment of transcription machinery and assist promoter DNA melting. (b) Nucleosome destabilization in the gene body is a mechanism to achieve high elongation efficiency. Positive supercoiling in front of transcribing Pol II propagates faster than the rate of elongation. The resulting torsional stress weakens the contacts between DNA and core histone by promoting H2A/H2B dimer eviction from the nucleosomes. Chromatin responds to DNA supercoiling by confinement of gene domain. This confined state of chromatin enhances the frequency of interaction among distal transcription regulators and Pol II.

Genome-wide studies show that TFs bind to sites that are largely cleared of nucleosomes [43]. Nucleosomes are dynamic structures that must be modified for the precise control of gene expression [44]. Current evidence favors active nucleosome eviction or depletion by factors like SOX2 and OCT4 and/or by multiple nucleosome remodeling factors [45,46]. Common thinking is that nucleosome remodeling provides a mean of regulating genomic accessibility [29], allowing DNA binding proteins to gain access to their otherwise nucleosome-protected target sites. As discussed above, DNA accessibility alone is not the only determinant of DNA–protein interaction. Another key factor is the affinity between DNA and regulatory factors that may be modified by mechanical forces acting on DNA. What forces act on DNA prior to TFs binding? Core histone rearrangement by all Snf2p-related nucleosome remodelers and/or acetylation by factors such as p300/CBP generate torsional tension in DNA by un-restraining negative supercoils held by nucleosomes (Figure 1) [26,47–49]. Torsional stress generated by ATP-driven remodelers is high enough to drive transition from B-DNA into unusual DNA conformations, so-called non-B DNAs [2]. It has been shown that the silent CSF1 promoter is activated by the chromatin remodeling enzyme BRG1, which removes the nucleosome from the promoter and drives Z-DNA formation [27,28]. Z-DNA is then involved in locally preserving the remodeled state of the chromatin. All these considerations lead to a model where local changes in chromatin structure during transcription initiation, introduce torsional stress into the DNA which favors loading of TFs, RNA polymerase II recruitment and finally PIC formation (Figure 3, A). If this model is true, then the first punch of DNA torsional stress required for transcription activation could be created not only from nucleosome eviction but also from upstream transcriptional activity. Indeed, a classical study demonstrated that gene expression can be switched ON by the negative supercoiling diffusing from a nearby divergent promoter [50]. Later, this observation and additional studies led to the elegant hypothesis that widespread divergent transcription initiation in the mammalian genome is necessary to keep promoters under torsional stress to facilitate transcription factor binding and ultimately mRNA production [16,20,51]. In accord with this prediction, identification of promoters supporting divergent transcription in the mouse genome revealed a high level of TFs binding [43].

### Open complex formation

Once the PIC is formed, the promoter DNA is melted locally, and the template DNA strand is stabilized within the Pol II active site forming a transcriptional bubble 12–15 base pairs long [52–54]. DNA melting is favored by the protein complex TFIIH. The XPB subunit of TFIIH translocates on double helix away from the PIC. Because translocation is constrained due to interaction of TFIIH with other PIC components, the promoter DNA is rotated, leading to DNA un-twisting [52,53]. Torsional stress results in disruption of base pairing, creating a melted DNA bubble that is fed into the Pol II active site. Thus, the formation of an open complex is based on the DNA mechanical constraints. However, this mechanism is not universal [55], and the degree of dependence on TFIIH for DNA opening varies for different promoters. Promoters that are prone to melt easily in response to negative supercoiling can initiate transcription even without TFIIH [54]. It has been predicted and recently shown that localized disruption of base pairing is widespread in supercoiled DNA [56,57], suggesting that this might be exploited as a regulatory mechanism in open complex formation. At the unstable promoters, DNA strand separation is spontaneously nucleated in under-twisted regions [54] explaining why the translocase activity of TFIIH is not required at certain promoters and may be circumvented if promoter DNA is supercoiled [36,58]. Taken together, these considerations highlight that the mechanical features of the DNA are important for open complex formation (Figure 3, A), a regulatory step in gene transcription [54,59].

### Early elongation and pol II promoter-proximal pausing

Once the open complex is formed, the Pol II catalyzes the formation of nascent RNA, a step known as early elongation. During this step, the transcriptional machinery still has tight contacts with promoter DNA and TFs [53]. Instead of polymerase moving forward along the DNA, downstream DNA is pulled into the early elongating complex, causing extension of transcription bubble [60,61]. Further progression of RNA polymerase requires promoter clearance. This necessitates the collapse of the upstream part of the extended transcriptional bubble, resulting in the abrupt reannealing of the two DNA strands [60,61]. Only about 15 base pairs remain melted in the Pol II active site. If DNA is negatively supercoiled, DNA can undergo spontaneous strand separation, exposing the two strands in a single-stranded bubble [2,57]. When the two strands of the bubble reanneal, supercoiling is released back into the surrounding DNA domain (Figure 1). Thus, the collapse of extended transcriptional bubble imposes high torsional stress on promoter. While the exact molecular mechanism driving promoter clearance is currently unknown [62], it is reasonable to propose that the high level of DNA supercoiling released on the promoter DNA drives extensive reorganization of both promoter and elongating complexes enabling promoter escape. Keeping the promoter under torsional stress might also be a prerequisite for the next round of transcription initiation [20,51].

Pol II escaped from the promoter produces a short, nascent RNA before it usually pauses 50 to 100 bp downstream from the transcription start site (TSS) [62]. This promoter-proximal pausing is considered as a rate-limiting step in the regulation of transcription [63]. A variety of factors converge to establish paused Pol II and mediate its release. In current models, the sequence of RNA-DNA hybrid, DNA elements around paused site, stability of nucleosomes and different positive and negative elongation factors such as NELF, DSIF, and P-TEFb can each influence the efficiency of nucleotide incorporation and pausing [62]. Early *in vitro* studies have shown the importance of Top1 in PIC assembly and transcription initiation [64,65]. As the catalytic activity of Top1 is not required for its role in initiation, it has been suggested that Top1 plays the role of an architectural factor, stabilizing bent or irregular DNA structure within the PIC [64]. Psoralen-based mapping of DNA supercoiling near promoters of a human cell line revealed that paused genes have much higher level of negative supercoiling at their TSS compared with elongating genes [66]. This might suggest that even if Top1 is localized in the vicinity of the paused Pol II, it cannot exert its ability to relax torsional tension. Torsional stress generated during early elongation can inhibit Pol II translocation by increasing Pol II stalling frequency and/or duration or by supporting inhibitory architecture within the early elongation complex. Single-molecule assays revealed that bacterial RNA polymerase may be stalled by torsional stress that accumulates both downstream (overtwisted) or upstream (undertwisted) DNA regions [67]. It has also been shown that the pausing of RNA polymerase triggered by the torsional stress could be relieved upon release of the opposing force [67]. Although the effect of supercoiling on stalling eukaryotic Pol II is still unknown, *in vivo* experiments suggest that excessive torsional stress does inhibit Pol II translocation [68,69]. It has been proposed that the pause and release into productive elongation are established by the specific DNA supercoiling balance at promoters [70].

A recent study using human HCT116 cells has shown that Top1 was catalytically inactive when associated with the early elongation complex before pause release, but active when binding with elongating complexes, demonstrating a role for Top1 in regulating Pol II promoter proximal pausing [71]. During this step, a positive feedback loop is established between Pol II and Top1 based on their physical interaction. Upon phosphorylation of the Pol II carboxy-terminal domain, the DNA relaxing activity of Top1 is enhanced and this, in turn, promotes pause release. This suggests that the low activity of Top1 at paused Pol II is not sufficient to remove the torsional stress that inhibits elongation, thus contributing to efficient pausing. After pause release, phosphorylated Pol II stimulates Top1 activity to remove mechanical impediments on Pol II translocation and promote productive elongation [71].

### Elongation

Pol II released from pausing into productive elongation is a highly processive enzyme. At full speed, it can translocate up to a remarkable 5 kb per minutes [63]. The main obstacles for efficient elongation are thought to be nucleosomes [72,73]. While traveling through a nucleosome array, the transcriptional machinery must pass a nucleosome every few seconds. However, early *in vitro* studies revealed that even a single nucleosome can impose a strong barrier for a translocating polymerase, highlighting the importance of mechanism/s allowing effective elongation *in vivo* [72]. Single-molecule studies have provided unprecedented clarity in examining the structural dynamics of nucleosomes [74]. It has become clear that nucleosomes take spontaneous excursions between wrapped and unwrapped DNA states displaying dynamic ‘breathing’ [75]. Pol II does not actively break contacts between histone core and DNA but waits until short stretches of DNA transiently unwrap from the core histones and then advances until the nucleosome is finally upstream of the translocating polymerase [76,77]. This ratchet-like moving through DNA of a nucleosome can be promoted by a variety of seemingly synergistic factors such as histone marks that loosen DNA-histone contacts, elongation factors, chromatin remodelers and underlying DNA sequences.

Experimental advances illuminated the role that DNA mechanical constraints could have in regulating nucleosome stability [73,78,79]. Torsional stress might change the conformation of the DNA in such a way that it becomes refractory to tight interaction with histones. It has been shown that single-stranded DNA breaks present in the non-template strand strongly affect the rate of transcription through the nucleosome but not the rate of transcription of naked DNA [73]. Nicked DNA cannot be supercoiled; thus, preventing the buildup of local torsional stress which is required for nucleosome destabilization and Pol II passage through the nucleosome [73]. Single-molecule study of nucleosome assembly on topologically constrained DNA has shown that nucleosome formation is inhibited by DNA positive supercoiling [78]. The nucleosome consists of an octameric protein core, formed by central H3/H4 tetramer flanked by two H2A/H2B dimers [3]. In studies that examine the effects of torsion on preassembled nucleosomes, even moderate positive torsion led to almost complete loss of H2A–H2B dimers from the nucleosome core, suggesting a possible mechanism for loosening the nucleosome barrier [79]. Indeed, the eviction of one H2A/H2B dimer results in unwrapping about 40 base-pairs of DNA which decreases nucleosome stability [80]. At physiological ionic strengths a nucleosome can block Pol II elongation, however with increasing ionic strength Pol II can bypass this block. With these conditions, *in vitro* transcription causes the loss of a single H2A–H2B dimer [81]. Therefore, it seems that positive torsional stress induces nucleosome destabilization by dimer eviction. *In vivo*, the factors that increase the torsional stress experienced by DNA favor nucleosome destabilization, whereas factors that decrease topological stress favor nucleosome stabilization [82,83]. Mapping of unwrapped nucleosomes genome-wide revealed that inhibiting topoisomerase activity *in vivo* increased unwrapping, whereas reducing Pol II elongation decreased the unwrapping of nucleosomes, specifically within promoter-proximal regions [82]. This argues that the positive torsional stress, generated in front of a transcribing Pol II, induces the loss of core histone-DNA contact to facilitate transcriptional elongation (Figure 3, B).

In *Drosophila* cells, transcription-generated positive torsional stress preferentially drives loss of contacts between DNA and promoter-distal H2A/H2B [82], mirroring early *in vitro* experiments [84]. What is the reason for the asymmetrical nucleosome destabilization? The intrinsic bendability of short DNA fragments from the genome of *Saccharomyces cerevisiae* were analyzed by ‘loop-seq’ assay [85]. As the nucleosome assembly requires extensive DNA bending [86], differential bendability across a nucleosome could potentially dictate which region of the nucleosome is destabilized first under positive torsional stress. The authors found that DNA at +1 and +2 nucleosomes has higher intrinsic bendability on the promoter-proximal face than on the distal face of the nucleosome [85]. This observation suggests that Pol II overcomes a nucleosome-imposed barrier better when promoter-distal face is destabilized by torsional stress. Interestingly, if the analyzed sequences were altered to use alternative codons for the same amino acids, the characteristic bendability pattern was lost. Thus, DNA mechanical properties dictate the asymmetry in nucleosome destabilization required for efficient elongation *in vivo*. These properties are under selective pressure to preserve nucleosome response to the torsional stress [31].

In principle, the diffusion of torsional stress through the chromatin should dynamically affect the organization of chromatin. Simplified modeling suggested that the wavefront of the altered chromatin progresses ahead of an elongating Pol II ~10 times faster than the rate of elongation [87]. This model is based on the ability of a nucleosome to adopt different entry and exit linker DNA configurations under a small positive torsional stress [88–90]. Nucleosome conformational changes help smooth the elongation process by buffering torsional stress experienced by DNA [91]. This wavefront is expected to stop at barriers which prevent torsional stress diffusion, such as insulators and boundary elements. A pioneering study of chromatin architecture at the Hsp70 gene of *D. melanogaster* provides evidence for rapid nucleosome disruption across the entire gene within 30 after activation, much faster than the rate of Pol II transcription [92]. The nucleosome disruption extends beyond Hsp70 and stops at insulating boundary elements. Subsequently, direct measurement of DNA torsional stress across genome of *D. melanogaster* revealed that accumulation of torsional stress results in increased nucleosome turnover, providing direct evidence for an *in vivo* influence of DNA torsion on nucleosome dynamics [69].

### Enhancer-Promoter communication

Large-scale chromatin movements have been shown to depend on transcription and topoisomerase activity, implicating DNA supercoiling in 3D dynamics of the genome [93]. Recent progress in super-resolution imaging combined with single-nucleosome tracking promises to improve our understanding of this phenomenon [94–96]. Contrary to the common view that transcribed regions are more open, in human cells, transcription activation resulted in threefold more confinement of an mRNA-producing gene domain within minutes [95]. This confinement is consistent with the response of chromatin to the axial rotation of DNA observed in single-molecule experiments (Figure 3, B). Rotations that produce positive supercoiling dramatically compact the chromatin fiber [89]. Further, constrained mobility was an immediate response to transcription initiation and was not dependent on ongoing polymerase elongation. Single-nucleosome imaging in human cells revealed that active Pol II constrains chromatin movement globally. Rapid inhibition or depletion of Pol II was able to release the chromatin constraints [96]. Similar spatial compaction and temporal stabilization of chromatin in response to immediate transcription has been reported in *Drosophila* embryos [94]. Cross-correlation between population averaged genome-wide studies and global imaging of chromatin dynamics in single cells suggests that local Pol II activity is sufficient to alter the chromatin environment even at a distance. This process likely involves dynamic positive supercoiling acting as a means for signal propagation between TSS proximal regions and regions far downstream in gene bodies. Discovery of GapR, a bacterial protein that preferentially recognizes overtwisted DNA can be used as a tool for the imaging of positive DNA supercoiling in the gene body, and might provide direct evidence to this suggestion [97]. In fact, the GapR-sequencing in yeast already has revealed that positive DNA supercoiling accumulates near the 3’ ends of transcribed genes and correlates with the transcriptional activity of the gene, thus, confirming the prediction of the twin-supercoiled domain model [97]. Lastly, GapR sequencing results are in line with psoralen intercalation studies in *D. melanogaster* cells [69].

**Figure 4.**
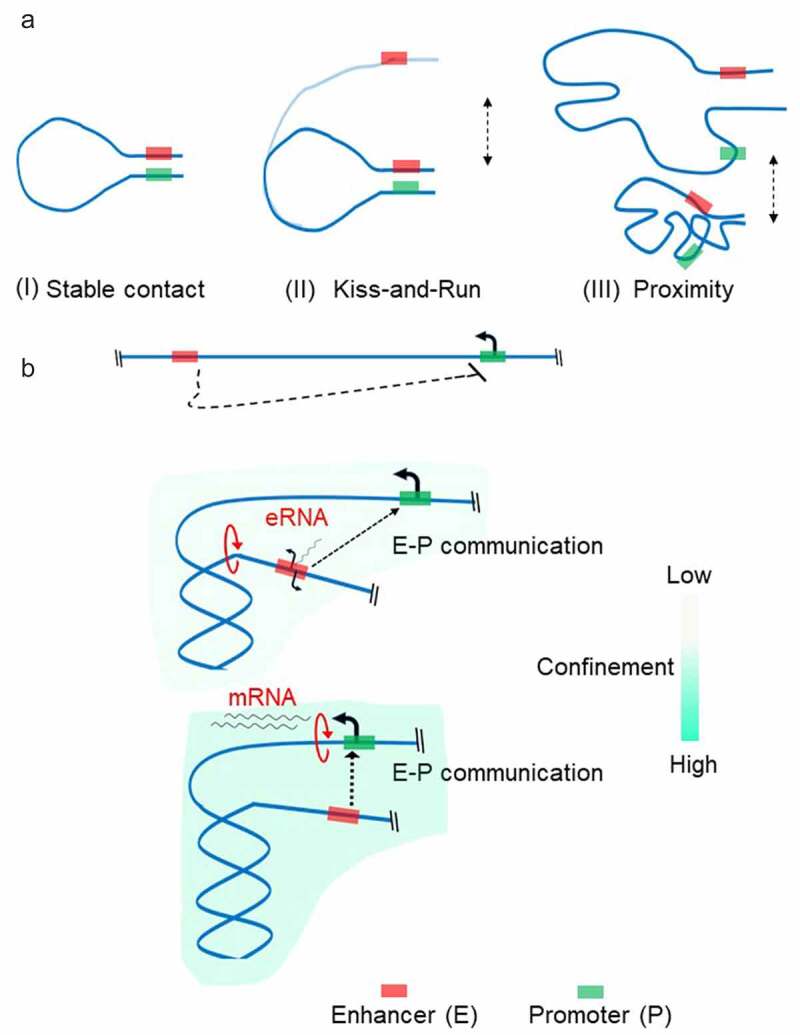
**Enhancer-Promoter** (e-p) **communication** There are at least three models by which an enhancer-promoter communication is established (a). The classical model (i) where transcription factors bind within their target enhancer and promoter form a stable complex between enhancer and promoter to stabilize the chromatin loop. In ‘kiss-and-run’ model (**II**), only transient physical contact between enhancer and promoter is required to regulate promoter activity. In proximity model (**III**), the enhancer communicates with the target promoter in a distance-dependent manner through the high local concentrations of transcription factors established by ‘hub’ or ‘condensate’ formation. Enhancer (red rectangle) is located far from the promoter (green rectangle) and may not communicate in a linear scale (**B**, Top panel). Bidirectional transcription at the enhancer region induces positive torsional stress resulting in confinement of region between enhancer and promoter (**B**, Middle panel). Enhanced spatial exploration of chromatin fiber promotes establishing functional E-P communication. Upon activation of the targeted promoter, the enhancer transcription is no longer required (**B**, Bottom panel). For clarity, transcription factors and Pol II complex have been omitted.

Confinement of the transcribed locus due to positive supercoiling is expected to increase the frequency of direct interaction between distal transcription regulators and Pol II, bringing them into proximity. The question is whether cells evolved to use these phenomena for regulatory reasons. The rapid development of Hi-C methods during the last decade has enabled the detection of loci in physical proximity on a genomic scale [8]. Enhancer-promoter (E-P) interactions were detected as local loops in Hi-C maps. Although sometimes the E-P interaction is established over 100 kb distance, on an average, enhancers map ~10 kbs to their targets in the mammalian genomes [7]. Specific transcription factors bind enhancer regions and recruit Mediator, a multi-subunit protein complex generally required for transcription [98]. The mediator then contacts the PIC assembled at a promoter to activate specific gene expression programs [98]. Due to the long separation between enhancer and promoter, the chromatin fiber can display random movements which can become an energetic barrier impeding the establishment of a productive communication. The most popular model of E-P communication is the looping of the intervening DNA to juxtapose the enhancer and the target promoter (Figure 4, A). Different models of functional loop establishment have been proposed: linking promoter and enhancer through protein interaction; Pol II translocation; or loop extrusion driven by cohesin complexes [7]. However, the direct visualization of enhancer–promoter interaction at the single-cell level has shown high variability between cells suggesting that stable loop formation might not be required for gene activation [6,10,11]. This evidence had led to the models of E-P communication where the requirement of physical proximity of enhancers with their target promoters rather than their physical association is most important for a productive communication (Figure 4, A) [7,99,100]. The mechanism of proximity establishment is currently unknown and subject of speculation [101].

Active enhancers are often transcribed [102,103], but the role of enhancer transcription remains a matter of debate [104]. Functional requirement for transcription at enhancers has been recently shown in activated mouse B cells [105]. The authors found that 50% of paired enhancers–promoters are coordinately expressed, and that E-P communication cannot be established if enhancer transcription is switched off, regardless of other features of the active enhancer. The reactivation of the enhancer transcription instantly activated the transcription from the target promoter. More importantly, enhancer transcription was dispensable if E-P interaction was established and the target promoter was already switched on, at least short term [105]. The direct effect of gene activation on the E-P communication came from the direct visualization of the gene regulatory element and transcription at the single-cell level in *Drosophila* embryos [94]. Transcription from the target promoter endorses temporal stability of the proximity between the promoter and enhancer as well as spatial compaction, demonstrating how the act of transcription can dramatically affect the 3D topology of chromatin.

Thus, we propose here that a two-step pathway might occur when E-P communication is dependent on enhancer transcription (Figure 4, B). First, translocation activity of Pol II at the enhancer introduces positive torsional stress and results in spatial compaction of genomic region between enhancer and promoter. This increased compaction favors enhancer–promoter proximity for sustained transcription. The second step is activation of the targeted-promoter transcription. Consequently, the function of spatial compaction is transferred to Pol II elongating along the gene. Once the second step is reached, enhancer transcription may no longer be required. Indeed, analysis of a wide range of cell types has shown that a rapid burst of enhancer transcriptional activity is frequently followed by its fast return to baseline [103].

## Transcription on a megabase scale

**Figure 5.**
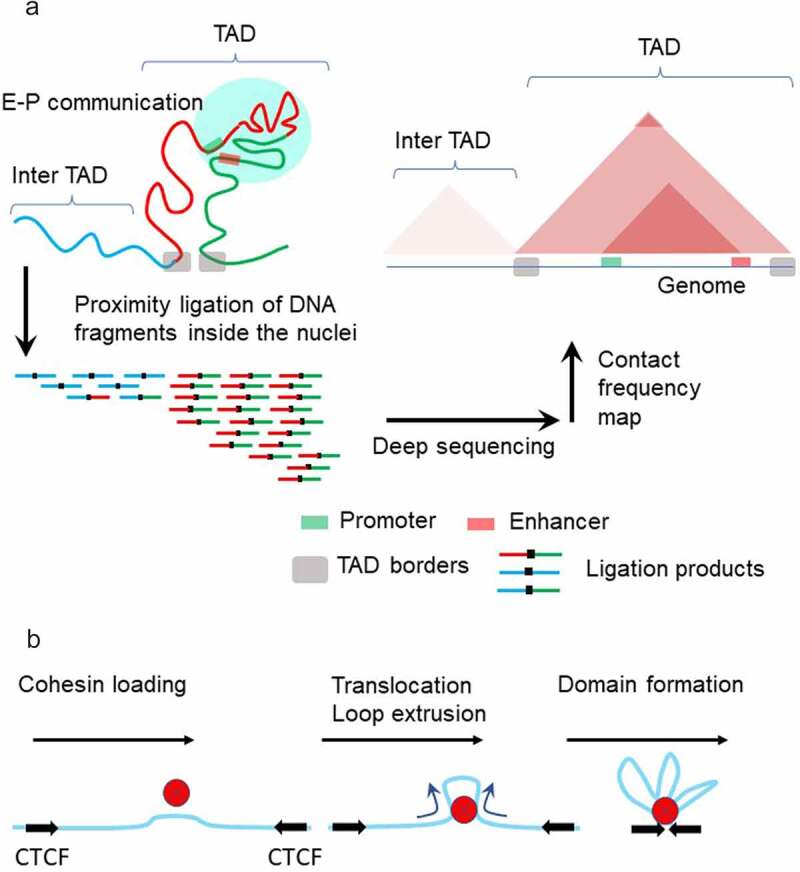
**Model of chromatin topology** Chromatin fibers are partitioned into topologically associating domains (TADs) (**A**, left). TADs in the genome are detected by Hi-C method. First, cells are fixed by formaldehyde treatment, which crosslinks chromatin segments that are in proximity. After digestion with restriction enzyme(s), DNA fragments are re-ligated. Proximity between chromatin segments results in the higher incidence that fragments are ligated together (**A**, left). Deep sequencing of the ligated fragments and mapping sequencing reads on the genome enables genome-wide identification of contact frequencies among different genomic loci (**A**, right). TADs preferentially self-associate to create discrete structural blocks which appear as triangles in the Hi-C map. Within TADs, sub-domains with higher contact frequencies are formed, often representing the confinement of region between enhancer and promoter. Chromatin loop extrusion is mediated by cohesin and CTCF proteins (b). Cohesin loads on DNA and begins translocating along DNA, resulting in loop extrusion. This process halts when cohesin encounters CTCF molecules, forming a chromatin loop with cohesin and CTCF present at its base.

In the current paradigm, miswiring of enhancers to non-target promoters is prevented by the formation of large-scale, up to Mbs in length, chromatin loops and TADs [6,8,106]. Methods of chromosome conformation capture have shown that these structures impose physical proximity on gene clusters and gene regulatory elements which is thought to represent an important feature in controlling gene expression (Figure 5, A) [8]. TADs and loops boundaries are enriched with cohesin complexes residing inside the domain, topoisomerase 2 enzyme residing outside the domain and the architectural factor CTCF at base of the domain [107].

The most accepted mechanism of domain formation is loop extrusion upon entrapment of the chromatin fiber by cohesin (Figure 5, B). Extrusion proceeds until cohesin becomes stalled at the CTCF-bound sites. However, the mechanism that drives cohesin translocation on the extruded loop is unknown [108]. Single-molecule experiments have demonstrated that cohesin and its mitosis-specific analog condensin can extrude DNA loops that are kilobases in length. Importantly, loop-extrusion requires adenosine triphosphate (ATP) hydrolysis both *in vitro* and *in vivo* [109,110] and is sensitive to mechanical forces. Extrusion is inhibited at <1 pN stall force [109], which is an order of magnitude smaller than stalling forces for RNA polymerases [111–113]. Cohesin is also able to compact DNA in the absence of ATP, however it has a strong preference for compacting positively supercoiled DNA [114]. Indeed, it was shown that cohesin recruitment *in vivo* is enhanced in regions where positive supercoiling is generated: ahead of transcribed Pol II [97] or in front of progressing DNA replication forks [115]. Conceivably, the mechanical forces acting on transcribed DNA might be a key modulator of loop-extrusion, favoring or inhibiting domain formation [116]. While the contribution of transcription to chromatin folding is still debated, increasing evidence points to a connection between Pol II binding to chromatin and TADs/loops formation [96,117,118]. Transcription and topoisomerase activities are involved in maintaining a steady state profile of torsional stress within the chromatin regions [66,69,119]. The discovery that TOP2 interacts with CTCF and cohesin at the borders of chromosomal domains led to the hypothesis that the DNA supercoiling generated by transcription along with TOP2 help to form chromosomal domains [120,121].

**Figure 6.**
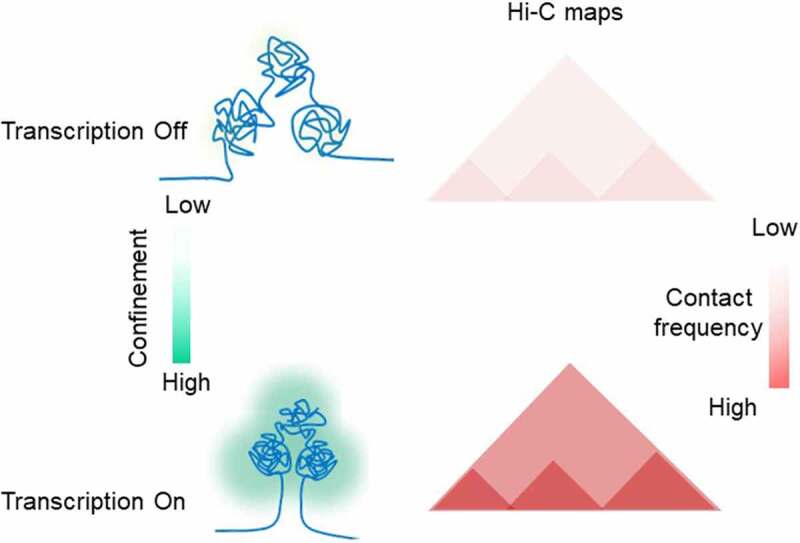
**TADs formation** Schematic showing active transcription supports TAD formation in DNA supercoiling dependent fashion.

Two recent studies shine new light on how transcription modulates proper domain formation in the chromatin. In the first study, Pol II was acutely depleted in human DLD-1 cell line to assess its contribution to genome folding [122]. Hi-C analysis of chromatin folding on G1-sorted cells after extended depletion (14 hours) revealed only a mild disruption of TADs. Hi-C data from cells depleted of Pol II for 2 hours did not reveal any changes in 3D genome organization. However, when cells were synchronized in G2, and Pol II depleted, then released via mitosis into G1, a strong and widespread destruction of domain structures was observed. The model predicting a role of DNA supercoiling in TADs formation envisages that in the absence of transcription, less cohesin will be translocated toward the CTCF-bound sites [121]. Indeed, in Pol II depleted cells entering G1 phase, the cohesin signal at CTCF sites was significantly reduced. These results indicate that transcription is involved in reestablishing chromatin folding during mitotic exit and hence suggests that transcription-generated DNA torsional stress is partnering with cohesin and CTCF in the loop extrusion process (Figure 6).

In another study, chromatin loops decorated with cohesin and Pol II were visualized in human HeLa cells [123]. Depletion of the cohesin-releasing factor WAPL (a human ortholog of the *Drosophila* wings-apart like protein (Wapl)) caused chromatin condensation in interphase cells through the process of enhanced loop extrusion [124]. Upon WAPL knockout, transcription inhibition disrupts loop formation and alters the cohesin distribution. Remarkably, a similar outcome was observed when topoisomerases were inhibited. Because both scenarios lead to a net imbalance in the level of supercoiling, the findings indicate that fine-tuning of supercoiling in chromatin favors loops extrusion (Figure 6). To validate this hypothesis, the authors probed the levels of DNA supercoiling by combining psoralen assay with imaging approaches. While high levels of psoralen intercalation were detected in the WAPL deficient cells compared to normal cells, upon inhibition of transcription and topoisomerase activity, the incorporation of psoralen was strongly decreased. Following assumptions derived from dynamic simulation studies [121], the authors concluded that cohesin loop extrusion is promoted by the accumulation of transcription-generated negative supercoiling. Although possible, these experiments could not clearly indicate whether positive or negative DNA supercoiling controls cohesin activity. Unfortunately, very high concentrations of biotinylated psoralen were used in these experiments. Under these conditions, psoralen binding to DNA mostly reflects chromatin structure at the nucleosome level [125,126]. Two orders of magnitude less psoralen would be required to assess DNA torsional stress [127,128]. Nevertheless, the high affinity of psoralen for DNA in cells with increased loop extrusion, suggests global nucleosome destabilization, as expected for a positively supercoiled genome [69,79].

## Mechanical epigenetics

DNA supercoiling may be considered as an epigenetic mark, playing an important role in establishing cell identity [12]. However, the mechanism of its inheritance, how the topological information from parental cells is transmitted to daughter cells, has been debated. Two main hypotheses for the supercoiling-based mechanism of transmitting cellular memory through mitosis have surfaced: conservation of the distribution of nucleosomes constraining supercoiling in chromatin domains [129], and the preservation of non-B DNA structures near the TSS of genes that are scheduled for activation in G1 [130].

A highly significant study has shown now that cell fate or memory is tightly linked to positive supercoiling acquired during transcription in the previous cell cycle. If the genomic distribution of positive supercoiling is disturbed, the next cell cycle is impaired. The study shows that during mitotic transcription [131], Pol II and Top1 coordinate their activities [132]. Deregulated Pol II-Top1 coordination or acute degradation of Top1 cause mitotic defects, cell cycle delays, and impaired transcription in the following cell cycle. Importantly, impaired Top1 activity results in a strong increase of negative supercoiling near genes active in mitosis, indicating that DNA supercoiling in mitotic chromatin is a critical dominant of cellular fate. Previous reports pointed to the importance of supercoiling in the mitotic function of condensin complexes [133]. Condensin is a close analog of cohesin and is also able to extrude chromatin loops, which is thought to be important for mitotic chromosome formation. Condensin-mediated loop extrusion favors positively supercoiled over negatively supercoiled and relaxed DNA [109]. This preference indicates that positive supercoiling acquired in TADs and loops during the cell cycle is important for appropriate condensin function. If the required level of positive supercoiling is lost because of excessive annihilating negative supercoiling generated by mitotic transcription, the transition to the new cell cycle is delayed and the daughter cells experience a ‘temporal amnesia’ [132].

## Supercoiling – belief versus reality

Taken together, our interpretation of the available data is that: transcription and 3D genome organization are influenced by negative DNA supercoiling across short scales and by positive supercoiling at the long scales. Negative supercoiling operates at 1–10 kbs-scale [66,69,119] mostly near the promoter regions regulating initiation (reviewed here) and fine-tuning of transcription (reviewed elsewhere [2,20,70]). Positive supercoiling operates at longer 10–100 kbs-scale regulating (1) nucleosome conformational changes that help to smooth the elongation process by buffering mechanical stress and facilitate H2A/H2B dimer loss for nucleosome destabilization; (2) physical proximity between enhancers and their target promoters; (3) the construction of TADs by loop extrusion; and (4) propagation of transcriptional memory from parental to daughter cells.

The long-range operation of positive supercoiling is explained by single-molecule experiments which show that the chromatin fiber could accommodate DNA overtwisting without a global buildup of torque [88,89,91]. Without the accumulation torque, positive supercoiling escapes the relaxation activity of the most abundant cellular topoisomerase Top1, a stress-sensitive enzyme that quickly responds to changes in DNA twisting [134]. Thus, the plasticity of chromatin to positive supercoiling allows for much further diffusion of supercoiling through the chromatin fiber. The topological plasticity of chromatin seems highly relevant to TADs/loop formation based on the single-molecule experiments [91,135]. Curiously, an *in vivo* study using yeast mini-chromosomes shows that positive supercoiling promotes the approximation intramolecular DNA segments [136], a feature in accord with TADs/loops on Hi-C maps [8]. Unlike positive supercoiling, unwinding of DNA in chromatin is directly converted to under-twisting of linker DNA [89,91] which is efficiently relaxed by Top1, limiting diffusion of negative supercoiling through the chromatin fiber [66]. However, in the current literature, the tendency is to consider more negative supercoiling as an active player in regulation of transcription and 3D chromatin structure, while the existence of positive DNA supercoiling is considered equivocal. The recent studies proposing supercoiling as the driving force for 3D genome re-organization are often based on the loose assumption that only negative supercoiling is persistent in genomes, while positive supercoiling is rapidly and/or preferentially removed by DNA topoisomerases [121,123,137–139]. The basis for this assumption arises mainly from the over-interpretation of studies performed in Levens’ and Roca’ laboratories [66,71,140] as well as over-simplification of psoralen-based experiments.

(1) We would like to take this opportunity to clarify that none of our work shows that Top1 localizes in front of Pol II and preferentially relaxes positive supercoiling as too often is suggested. Catalytic activation of Top1 during pause release does indicate that torsional stress contributes to efficient pausing by creating mechanical impediments [71]. However, this stress might equally derive from either positive or negative supercoiling. Given the high topological plasticity of chromatin to the positive supercoiling, our current thinking is that positive supercoiling has very little impact on the processivity of the Pol II and is even required to establish an elongation ‘friendly’ environment [19,88,89,91]. Only transcriptions through long genes can generate enough positive supercoiling to necessitate special mechanisms to remove it [141]. At the same time, high negative supercoiling detected near the sites of pausing suggest that this supercoiling reinforces Pol II pausing and so needs to be adjusted by activated Top1 at to commence productive elongation [66]. In line with this rationale, it has been shown that impairment of the bromodomain chromatin factor BRD4, which serves as mediator for Top1 activation [71], leads to an accumulation of RNA:DNA hybrids (R-loops) [142]. R-loops formation could be dangerous to the genome, perhaps causing lethal DNA damage [143]. They are also known to be directly sponsored by aberrant buildup of negative supercoiling [144], stressing the importance of removing excess negative supercoiling.

(2) Roca’s group elaborated on the observation that transcriptionally active circular mini-chromosomes in yeast acquire high levels of negative supercoiling in the absence of Top1. Under variety of conditions, they concluded that Top2 removes positive supercoiling faster than negative, whereas Top1 relaxes supercoiling indiscriminately [140]. Although these results are solid, care must be taken to not over-generalize them to the linear genome organized by different architectural and mechanical constraints. Indeed, mapping positive supercoiling by GapR-seq assay in yeast cells has indicated that positive supercoiling is a persistent feature of the yeast genome, confirming all the predictions of the ‘twin-domain’ model [97].

(3) DNA supercoiling has been detected genome-wide primarily through binding of psoralen to DNA inside living cells (Figure 2, b). Since the difference between binding affinity of psoralen to different topological forms of DNA is small, statistically significant detection of positive versus negative supercoiling requires the application of complex mathematical analysis or deep sequencing to reduce noise in genome-wide studies [127]. The topological plasticity of chromatin further increases the difficulty of ascribing the psoralen-binding pattern to positively supercoiled DNA [89]. Despite the introduction of high levels of supercoiling in front of the elongating polymerase, only a fraction of it distributes into overtwisting of the double helix. Because psoralen affinity is lessened with DNA overtwisting, detecting positive supercoiling is challenging. Consequently, the conclusion that positive supercoiling is preferentially relaxed by topoisomerases *in vivo* has not been rigorously established [121,123,137]. Perhaps psoralen-based deep sequencing approaches may definitively reveal and map positive supercoiling genome wide [69]; this will allow comparison with newly developed GapR-seq assay [97] and potential decoding of DNA conformation-function in genome biology.

## Conclusion

Our understanding of the critical role played by DNA and chromatin mechanics in genomic transactions has only recently received its due attention. The role of mechanical effects such as DNA supercoiling in eukaryotes remained an unfamiliar theme for many years, with studies mostly restricted to bacteria and plasmid systems. However, recent evidence demonstrates that virtually all DNA-based processes both control and are controlled by dynamic forces and torques acting on DNA in the chromatin. DNA supercoiling is a mediator that connects genome functions to structural reorganization of the genome, which in turn feedback to regulate the functions themselves. Incredible progress in genome-wide detection of DNA torsional constraints, theoretical studies, and single-molecular experiments have led to the conclusion that DNA supercoiling is an epigenetic mark possessing many layers of feedback regulations. Yet, although many scientists working in the fields of DNA and chromatin topology recognize the importance of DNA mechanics to most fundamental nuclear processes such as transcription, 3D genome organization, and replication, overall acceptance by the broader community remains to be achieved. Many peer-reviewed papers attempt to explain genomic processes mainly through the static architecture of chromatin. These studies largely ignore the functional dynamics of chromatin and the role of DNA mechanical constraints. We argue that these studies will prove insufficient to explain the biology of chromatin until the physical characteristics and the dynamics of the material from which it is built are considered. Therefore, combined efforts of multidisciplinary studies are imperative to understand how DNA mechanics affect genome function and structure. We imagine that new tools will ultimately allow the visualization of DNA supercoiling in single cells with high spatial and temporal resolution. We hope that this review will help stimulate the curiosity and intellectual excitement of bright minds in different disciplines to join in the investigations of this exciting field.
